# Utilizing the determinants of healthy aging to guide the choice of social prescriptions for older adults

**DOI:** 10.24095/hpcdp.44.9.05

**Published:** 2024-09

**Authors:** Beth Mansell, Anne Summach, Samantha Molen, Tammy O’Rourke

**Affiliations:** 1 Healthy Aging Alberta, United Way of Calgary and Area, Calgary, Alberta, Canada; 2 Faculty of Nursing, University of Alberta, Edmonton, Alberta, Canada; 3 Sage Seniors Association, Edmonton, Alberta, Canada; 4 Faculty of Health Disciplines, Athabasca University, Athabasca, Alberta, Canada

**Keywords:** healthy aging, seniors, older adults, community health, frailty, referral

## Abstract

The age of Canada’s population is increasing, necessitating innovative methods and tools for assessing the needs of older adults and identifying effective health and social prescriptions. In Alberta, a community-based, senior-serving organization undertook the development and piloting of the Healthy Aging Asset Index, an assessment tool and social prescribing guide for use by a variety of professionals within the community. Tool development was rooted in medical complexity assessment and social work practice, and adhered to the determinants of healthy aging established by Alberta’s Healthy Aging Framework, which is based on the determinants of healthy aging published by the World Health Organization. Results from the pilot showed improvement in the functionality of older adults within the determinants over time, as they were supported in addressing areas of personal vulnerability. Adopting tools such as the Healthy Aging Asset Index can bring cohesiveness to the support that older adults receive across the care continuum and has the potential to shift the balance of care away from the health system and towards the community, thus improving the capacity of health systems and government to meet the needs of Canada’s older adults.

**Highlights**

- The Healthy Aging Asset Index (HAAI) is an assessment tool that can be used to guide social prescribing by a variety of professionals in the community.
- The determinants of healthy aging can be used to inform social prescriptions in different domains.
- The HAAI can support shifting care away from the health system and into the community, and improve the capacity of health systems.
- Further investment is needed to support the implementation of the HAAI and social prescribing pathways within community-based organizations.

## Introduction

The age of the Canadian population is increasing, along with the need to understand the factors that affect the ability of older adults to age well in community.^1^ Organizations must develop programs to respond to these factors. The world population is aging faster than ever before,^2^ and the ability of health systems to provide care to the older adult population is limited. Frailty is one of those factors affecting the ability of older adults to age well in community. Frail older adults require holistic models of care to optimize patient-centred outcomes and improve quality of life.^3^ In spite of this need, previous research has only examined how frailty could be screened for in medical contexts such as primary care, ambulatory care, assisted living, long-term care homes, acute care and critical care settings.3 Community-based organizations that employ a strengths-based approach to increase resiliency in older adults are uniquely positioned to identify and respond to frailty through social prescribing programs; new practices and policies should be geared toward community solutions to address frailty in systems outside of medicine.

In Alberta, more than one million people will be aged 65 or older by 2035.^4^ Older adults currently make up less than one-fifth of the population of Canada, yet they account for nearly half of all health care expenditures.^1^ Many older adults live with some degree of frailty, and there is a link between frailty and chronic diseases, especially when considering socioeconomic status. In Ontario, the prevalence of having five or more chronic diseases is 11% among low-frailty, 26% among medium-frailty and 44% among high-frailty groups.^5^ Increased frailty has been associated with lower neighbourhood-level income,^5^ and research has shown that social factors can be a predictor of hospitalization.^6^ These costs and challenges are adding pressure to health systems’ post–COVID-19 response, including long wait times for emergency and surgical care, difficulty reaching health screening targets in primary care (i.e. pap smears, mammograms, prostate screening) and the provision of on-time primary care services for simple tasks, such as prescription refills.

Given the current dramatic demographic trends and the inability of health systems to respond to the care demands of an aging population, there is a need to do things differently. A potential solution is to identify frailty early and address the social and nonmedical needs of older adults in locations other than physician offices and primary care settings, underscoring the notion of the right provider, in the right place, at the right time—an approach that systems planners have been pursuing to improve service efficiency and effectiveness.^7^ Providing care and addressing the needs of older adults living in the community can be complex, and appropriate tools and resources are required to support this goal, especially as the number of older adults living in the community increases due to demographic changes. Case complexity has been identified as a key contributor to increased health care use, and methods to identify those individuals who require targeted assessments to inform interdisciplinary interventions have been discussed in the literature.^8^-^12^ Social prescribing has been defined as “a means for trusted individuals in clinical and community settings to identify that a person has nonmedical, health-related social needs and to subsequently connect them to nonclinical supports and services within the community by co-producing a social prescription—a nonmedical prescription, to improve health and well-being and to strengthen community connections.”^13^^, p.9^ Assessing complexity and risk in older adults can identify opportunities to build resilience through options such as social prescribing and help to reduce downstream medical effects.^8^

For social prescribing to effectively connect older individuals to nonclinical supports and services, there is a need for a shared comprehensive (social and clinical) assessment that focusses on strengths, resiliency and positive change, rather than on deficits, and that facilitates integration of the services provided by voluntary and community organizations in collaboration with primary care.^14^ However, there is a lack of assessment tools that address social and clinical assessment needs for this population. This article describes the development of an assessment tool, the Healthy Aging Asset Index (HAAI), informed by the Healthy Aging Framework, and its potential use in community agencies to facilitate social prescriptions and address frailty. 

## Development of the Healthy Aging Asset Index

The HAAI was developed to facilitate a more in-depth, comprehensive assessment of older adults’ risk factors for functional decline, as there is a lack of clarity regarding how to respond to older adults who present with complex health and social needs. Ideally, anyone serving older adults within the community, where over 92% of older adults live and take recreation,^15^ could identify frailty and administer healthy aging assessment. An interprofessional team developed and sought feedback from a variety of stakeholders on the HAAI, which used common language to support early and efficient assessment and identification of clinical and social interventions. These interventions inform the development of personalized asset plans for healthy aging of older adults living in the community. Assessment and interventions are based on a social prescribing model embedded in an anti-oppressive, holistic approach to care. 

Using this approach acknowledges that health concerns of older adults may be exacerbated by the social conditions in which they live that are beyond their control.^16^ Assessment and planning are also based on the knowledge that older adults living in the community experience different levels of frailty—some are “minimally frail,” many are “moderately frail” and a very few are “severely frail,” categories established through use of the Clinical Frailty Scale.^17^ Individualized plans can optimize social prescribing to address social and medical complexity and direct both clinical and social prescriptions, a type of integrated social prescribing approach that is currently used only in a very limited capacity in Canada.

We conducted a review of the literature related to complexity in older adults and other underserved populations living in community. This review of both scholarly and grey literature identified several tools that address population complexity indicators. None of the tools included a focus on strengths instead of deficits, and all tools lacked comprehensive social assessment components, both of which are increasingly called for in the literature.^18^,^19^ Current approaches with older adults, such as anti-oppressive, person-centred social work practice, take a strengths-based approach that emphasizes the possibilities, capabilities and capacity of older adults. These strengths may be accumulated over years of life and optimized within a support network of family, friends and care providers. Identifying and working with what older adults bring to the care relationship fosters inclusion, validation and empowerment.^18^

Building on the example of an intake tool of an Edmonton-based nonprofit organization, the HAAI was developed to address polypharmacy, the occurrence of chronic conditions, and medical attachment, as well as a variety of social factors such as safety, economic stability and housing. It was critical, given that implementation occurred in the early days of the COVID-19 pandemic, that the tool could be used both in person and via a telehealth appointment, to facilitate ongoing support for older adults experiencing significant health care access barriers. In addition, the tool should be usable, understandable and reliable across health and social assessors. The resultant product included strengths-based language, focussed on a range of determinants of health, was easy to use, supported social prescribing and provided a quantitative score that could be incorporated into evaluation and reassessment plans.

The Healthy Aging Asset Index (HAAI) incorporates seven domains that align with the Healthy Aging Framework’s determinants of healthy aging.^20^ The Healthy Aging Framework (HAF) is a tool that can be used to articulate, organize and communicate the work of senior-serving organizations, and is based on the determinants of healthy aging (DOHA) established by the World Health Organization.^21^ The DOHA are the domains of the framework under which all work is organized. Below the DOHA are service areas, followed by specific activities, outcomes and impacts. The HAF can be used for strategic planning, priority setting, evaluation within organizations and coordination across the sector. DOHA play an integral role in the adversity, challenges and vulnerabilities individuals face as they age.^16^ The DOHA listed in the HAF were adapted to create the seven domains of the HAAI: physical health, personal well-being, mental health, social support, physical environment, safety and security, and social engagement. 

Structuring the HAAI according to Alberta’s HAF allows an assessor to easily identify areas in which an individual could benefit from social prescribing. This is accomplished through a series of questions for each of the DOHA; each determinant is scored out of a total of four points. For example, within the physical environment determinant, questions address housing, poverty and transportation: “Do you have a safe place to live, is it affordable, and do you want to continue to live there?”; “Is it hard to make ends meet each month with your current income?”; and “How do you normally get to appointments/shopping?” Based on the responses, the tool will suggest interventions such as assistance completing an affordable housing application, assistance to access financial benefits and assistance to find transportation options. Individuals seldom require assistance across all domains, but frequently require targeted intervention to achieve specific goals such as increased social engagement, safer living arrangements, improved financial security or mental health stability.

Domains are scored and then combined as a total for each determinant. Scores of zero in a domain indicate a low level of complexity without any evidence of a need to intervene. Low-scoring domains on the HAAI indicate areas of strength and resilience. In domains with scores of one and above, clinical and social prescriptions are triggered to address factors contributing to vulnerability. High-scoring domains indicate areas of vulnerability, and the suggested clinical and social interventions are intended to stabilize older adults at risk of experiencing advancing frailty living in the community. The total score for the HAAI quantifies the overall resilience or asset status of the individual. This method aligns with the scoring guidance used by other complexity tools.^22^

Having a tool to direct assessment and intervention is critical for the spread of the process to nonclinical personnel, as HAAI scoring provides guidance regarding possible social prescriptions to enhance resilience in particular domains.^19^ An approach that optimizes the involvement of all professionals and addresses all determinants of health supports holistic care for older adults.^23^ In addition, scores in individual domains can assist with prioritization of needs within the total wellness picture for the individual, recognizing that the priorities of the assessor may not align with those of the older adult and will need to be negotiated collaboratively.

Clinical prescribing activities align with standard medical care and include prescription optimization, connection with primary care, mental and physical health supports, allied health connection and chronic disease management. Social prescribing activities are focussed on individual needs that are not immediately identifiable as “clinical” or “health-related,” though these social factors have a significant impact on the future health state and well-being of the individual.^23^ Assessors collaborate with older adults to create an asset plan that is acceptable to the individual and aligns with their health and wellness goals. Specifically, the most effective social prescriptions are those that are supported by workers embedded in the community who have built connections with diverse voluntary, community and social resources.^14^

## HAAI pilot methods

In the fall of 2019, funding was secured to pilot the HAAI with older adults who were identified as moderately frail. Older adults were screened using the Clinical Frailty Scale (CFS)^17^ to identify a quality improvement group (those who scored between 4 and 6 on the CFS) for whom additional assessments with the HAAI were completed. Older adults were recruited from incoming calls to the seniors association; callers were asked to complete a frailty screen, adapted from the Clinical Frailty Scale, identifying those who were minimally or moderately frail. These individuals were offered a call from the community connectors (link workers) to facilitate additional assessment with the HAAI. The HAAI was used as a comprehensive geriatric assessment to identify areas of resilience and vulnerability for this moderately frail group of older adults. The assessment was completed on admission to the pilot, and then repeated at 3, 6 and 12 months, allowing for tracking of scores over time. 

Older adults who were seen in person were also administered the Edmonton Frail Scale (EFS)^24^ as a comparative measure. The EFS scores were used to provide frailty context from a validated scale for those individuals who were screened with the HAAI.^24^ These data were limited due to the COVID-19 pandemic, making convergent validity determinations difficult. Formal content validity and interrater reliability processes were ongoing at the time of writing. During the pilot, administration of the tool by different types of professionals led to a depth of perspective during iterative tool development, and a more robust community and social services approach to implementation. Social workers were trained to administer both the CFS and HAAI to older adults living in the community via phone or in person, in essence working in a community connector role, which is well described in the social prescribing literature.^23^ Connectors took the lead in providing navigation support, directed by the individual’s primary care provider. 

Using the HAAI led the connectors to recommend increasing numbers of interventions over the course of the pilot to facilitate asset development for the older adult. This allowed nonmedical professionals to support connection to a wide range of interventions, contributing to conversations that gave older adults the agency to determine which diverse preventive and empowering supports were right for them. This practice, facilitated by the HAAI, builds on the concept of social prescribing, which is a structured system of referring people to a range of clinical and nonclinical services and leveraging the community-based sector to support an individual’s needs.

## Results

Following the HAAI pilot’s conclusion in 2022, a statistical analysis was completed to identify program and tool impacts on the functional level of participants. Data were available for 77 individuals aged 50 and older, across four time points from intake to final follow-up. The HAAI was used 210 times over the period of the pilot, as not all individuals completed the total number of follow-up assessments. The statistical analysis identified a need for standardization in category composition. Given that DOHA domains initially included two, three or four questions, completing comparative analysis between domains was difficult. When considering the effects of the HAAI program and the social prescriptions that were implemented, overall HAAI scores showed a statistically significant improvement after a 12-month period when controlling for age and gender. 

However, more important to consider is the scoring and improvement for each of the specific DOHA domains, as social prescriptions are targeted to the domain rather than the total HAAI score. Scoring on the tool is optimally as low as possible; the pilot demonstrated the highest scores in physical health and social engagement, identifying these domains as most problematic for participating individuals. Domains that seemed less concerning for the majority of participants were safety and security and personal well-being, a finding that is also supported in the literature.^25^

Given the type of data and number of older adults assessed, it was determined that a regression model fitted to panel data with random effects was the most appropriate. Due diligence suggested that additional statistical models be run using standard ordinary least squares regression, ANOVA and panel data with fixed effects. Panel data regression is more appropriate than ANOVA in this case because it allows for missing values. Random effects were used rather than fixed effects in order to test the significance of client age and gender. 

A statistically significant reduction in HAAI score was observed for clients at the 12-month assessment when compared to their intake score. In this analysis, the reduction is equal to a 4.1-point decrease (95% CI: 2.61-7.26; *p* < 0.001) in the total score when controlling for age and gender. 

Regression results for the overall HAAI score are presented below (Table 1). Time, age and gender together explain about 20% of the change in HAAI score within individuals, and just under 12% of the differences in HAAI score between individuals. When compared to the initial assessment, there was no significant change in the overall HAAI score at the 3-month or 6-month time points. When compared to women, men did not have a statistically significant different HAAI score. When compared to those aged 50 to 64 years, HAAI scores were not statistically different for those aged 65 to 74years, but they were significantly lower (by 6.51 points; *p*<0.01) for clients aged 75 years and older. 

**Table 1:** Summary of HAAI regression fitted to panel data with random effects, decomposed by domain

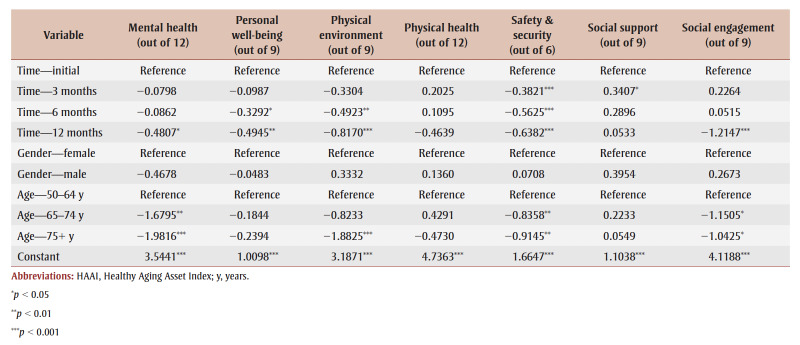

Analysis of pilot data also highlighted the domains that showed the most improvement over the course of the pilot, demonstrating statistically significant score improvements immediately in safety and security, 6-month improvements in personal well-being and physical environment, and 12-month improvements in mental health and social engagement. Physical health scores did not show significant improvement during the one-year pilot (Table 1). Declining social support scores are difficult to interpret, but may be related to launching the project at the onset of the COVID-19 pandemic.

Unlike traditional frailty or geriatric assessment tools, the HAAI pilot optimized the relational role of the community connector to ensure that the selected interventions aligned with the older adults’ current goals and assets, placing the older adult in the centre of the process, and in control. The use of modern social work approaches, such as solutions-focussed coaching and motivational interviewing,^26^ further enhances the HAAI not only as an assessment instrument, but as a collaborative tool that facilitates active partnership with clients. The HAAI goes beyond the immediate problem-solving model (i.e. treatment only), which is common in current frontline social work practice and outreach, to a practice philosophy that encourages greater relationship building and holistic and long-term wellness—a preventive approach for which many social work professionals have advocated.^10^ Future research efforts will target potential measurements of how HAAI use influences overall health and what the impact is on health resource use. Combining analysis with other measures such as hospitalization length of stay, quality of life, movement to settings offering higher levels of care, or emergency visits may provide relevant data to support expansion and public funding of social prescribing programs.

## Policy and program options

The HAAI, which was developed and intended for use across both the health and social sectors, expands the capacity of the health and community-based systems to identify older adults with complex needs seeking support, and clearly identifies potential options the older adult may have for resolving or addressing the issues that could, over time, contribute to increased frailty, increased societal cost and system pressures. Preventative, community-based care is an important priority for local, provincial and federal governments. 

As stated earlier, most older adults (92%) reside in private dwellings^15^ and require access to a network of community-based supports that will facilitate aging in place. Communities in turn require adequate funding for this network of supports, as well as a dedicated community connector or link worker to conduct comprehensive assessments using the HAAI. Though primary care clinicians recognize the impact that the DOHA have on older adults living in the community, they are ill-equipped to advise on resources within the community. Essential to the success of social prescribing models is knowledge of ever-changing community resources—expertise that cannot be expected of medical professionals and that social workers may not have capacity to keep up. The HAAI tool facilitates and organizes this knowledge for the link worker. The HAAI also provides an opportunity to link the DOHA domains with resource databases, such as the 211 information and referral service available in some provinces. 

Ensuring that there is seamless access and communication between health clinics and link workers will facilitate simple referrals from clinicians to the social prescribing structures that are in place. The HAAI tool, when used in conjunction with a screening mechanism such as the CFS, offers a clear pathway for social prescription and the associated interventions to be implemented. These tools can be used to identify and address frailty in systems outside of medicine, recognizing that frailty exists on a continuum and can be mitigated with a variety of social and clinical interventions. Social prescribing needs to be clear, accessible and simple to assess for, or it will fall to the bottom of the priority list of clinicians, despite its value.

Optimization of community-based approaches to the support of older adults is essential for the overall health of our society, and the long-term affordability of care for this population. Placing value on social needs such as affordable housing, financial security and food security will allow older adults to live healthier lives and decrease their use of the health system. This preventative action (concentrating on social needs) will not just shift a burden from one system to another, but will be more efficient and economical, since individuals will be more easily able to access resources and supports that meet these needs before having to access the health system. For this shift from health care to community to occur successfully, government funding is integral to supporting the development of social prescribing models and to ensuring that the tools and processes are supported by current research. The HAAI is shown to improve the process of assessment and intervention, and use of the tool can further advance social prescribing approaches in the community. Increased advocacy from the health sector, alongside the community-based senior-serving (CBSS) sector, is critical to promoting use of the HAAI and the link worker role, as has been demonstrated in other jurisdictions.^23^

The HAAI also allows community-based organizations to evaluate the efficacy of interventions using a common framework. During the HAAI pilot, assessments were repeated 3, 6 and 12 months post–program intake, which allowed for analysis of the impact of the social and clinical prescribing triggered during the process. This information is useful for informing policy and program decisions at all levels of government. For example, in the pilot, scores were highest in the physical health domain, yet did not improve significantly over a 12-month period. This could mean that the intervention options in the physical health domain were not meeting individuals’ needs, and that further funding in this area is required to develop new programs and services. Ideally, a larger implementation group would provide more robust data on which to base analysis, which in turn would support a greater understanding of the impact of prescribing on the trajectory of healthy aging.

## Barriers to implementation

Leveraging CBSS organizations as resources in a social prescribing model can prevent older adults from moving up the tiers of care and cost. However, a shift from health care toward community presumes a readiness among CBSS organizations that does not necessarily exist; the need to advance research, improve cross-sector collaboration and build system-level capacity in this area is evident. A systematic review examining facilitators and barriers of implementing and delivering a social prescribing service in the UK found that organizational readiness was a key facilitator to a successful social prescribing program.^27^ Organizations have to be “navigator ready,” and there is a need for a collaborative multisector approach to project management.^27^ In Alberta, it is recognized that the senior-serving sector is relatively uncoordinated, and organizations face challenges due to the lack of collaborative tools, streamlined referrals processes, sector leadership and common frameworks for action.^28^ However, work is underway through Healthy Aging Alberta that can be leveraged to promote the adoption of tools like the HAAI.

Capacity limitations within CBSS organizations also restrict the full implementation of assessment tools like the HAAI. Frontline staff are overworked due to the increasing demand for services, as they lack sufficient funds for staff and programming. This results in difficulties recruiting and retaining staff and makes it challenging to engage in systematic change management processes. In the UK, temporary staff contracts and staff turnover were found to be barriers to social prescribing implementation.^27^ When we shift care into community, more value must be placed on the importance of these frontline roles, as they facilitate relationship-based social interventions and have the potential to increase the positive impact on an individual’s health and well-being. CBSS programs and staff must be supported to the same degree as clinical interventions provided through medical professionals.

Currently, there is neither a systemic nor a systematic approach to the delivery of nonmedical services in community, including assessment and intervention, to support this shift from health care to community care. A lack of consensus on the best tool for assessing frailty has been noted as a barrier to implementing frailty assessments in clinical settings, despite the tools being adaptable to different settings.^29^ A systemic approach is crucial to the ongoing success and sustainability of a social prescribing model—one in which a link worker can make connections to necessary nonmedical services and assist social workers in meeting the needs of older adults. Assessment before social prescription was found to be one of three critical components for successful impact on the loneliness, health and well-being of older adults.^30^

Currently, social workers working within both medical services and community services are overwhelmed by growing caseloads of increasing complexity. Systematic efforts to address this challenge must include training of link workers, engagement of CBSS organizations and active recruitment of clinical partners to participate in graduated social prescribing pilots. Successfully demonstrating proof of concept within local community organizations should lead to more complex integration within health organizations, which would in turn reinforce the downstream health and financial impact of the model.

## Conclusion

The process of developing and piloting a healthy aging assessment tool based on the DOHA provided us with valuable insights into how social prescribing could be streamlined and leveraged within a CBSS organization. Older adults living in the community often require targeted support, aimed at facilitating higher levels of function within one of these key determinants of health, and the HAAI not only guides assessment of aging but also provides possible prescriptions to address identified areas of vulnerability. Consistent use of a tool such as this, across areas of practice and with diverse professionals involved in the care of older adults, will streamline assessment, service delivery and data collection. Ideally, the data captured from widespread use of the HAAI would support governmental decision making within the senior-serving sector, inform program investments and ignite innovative approaches to service delivery.

## Acknowledgements

Piloting of the HAAI tool was supported by a grant through Healthcare Excellence Canada. Additional support for the project was provided by the staff at Sage Seniors Association in Edmonton, Alberta. 

## Conflicts of interest

All authors declare no conflicts of interest.

## Authors’ contributions and statement

BM: writing—original draft, project administration, writing—review and editing.

AS: conceptualization, methodology, project administration, writing—review and editing.

SM: writing—review and editing.

TO: conceptualization, methodology, project administration, writing—review and editing.

The content and views expressed in this article are those of the authors and do not necessarily reflect those of the Government of Canada.
